# The Impact of Ageing on Diaphragm Function and Maximal Inspiratory Pressure: A Cross-Sectional Ultrasound Study

**DOI:** 10.3390/diagnostics15020163

**Published:** 2025-01-13

**Authors:** Toru Yamada, Taro Minami, Takahiro Shinohara, Shuji Ouchi, Suguru Mabuchi, Shunpei Yoshino, Ken Emoto, Kazuharu Nakagawa, Kanako Yoshimi, Mitsuko Saito, Ayane Horike, Kenji Toyoshima, Yoshiaki Tamura, Atsushi Araki, Ryoichi Hanazawa, Akihiro Hirakawa, Takeshi Ishida, Takuma Kimura, Haruka Tohara, Masayoshi Hashimoto

**Affiliations:** 1 Department of General Medicine, Graduate School of Medical and Dental Sciences, Institute of Science Tokyo, Bunkyo-ku, Tokyo 113-8510, Japan; 2 Medicine, Division of Pulmonary, Critical Care, and Sleep Medicine, The Warren Alpert Medical School of Brown University, Providence, RI 02903, USA; 3 Medicine, Division of Pulmonary, Critical Care, and Sleep Medicine, Care New England Health System, Providence, RI 02906, USA; 4 Department of Intensive Care Medicine, Aso Iizuka Hospital, Iizuka, Fukuoka 820-8505, Japan; 5 General Internal Medicine, Iizuka Hospital, Iizuka, Fukuoka 820-1114, Japan; 6 Dysphagia Rehabilitation, Department of Gerontology and Gerodontology, Graduate School of Medical and Dental Sciences, Institute of Science Tokyo, Tokyo 113-8510, Japan; 7 Department of Diabetes, Metabolism, and Endocrinology, Tokyo Metropolitan Institute for Geriatrics and Gerontology, Tokyo 173-0015, Japanaaraki@tmghig.jp (A.A.); 8 Center for Comprehensive Care and Research for Prefrailty, Tokyo Metropolitan Institute for Geriatrics and Gerontology, Tokyo 173-0015, Japan; 9 Department of Clinical Biostatistics, Graduate School of Medical and Dental Sciences, Institute of Science Tokyo, Bunkyo-ku, Tokyo 113-8510, Japan; 10 Department of Community Medicine (Ibaraki), Graduate School of Medical and Dental Sciences, Institute of Science Tokyo, Tokyo 113-8510, Japan; 11 Department of R&D Innovation for Home Care Medicine, Graduate School of Medical and Dental Sciences, Institute of Science Tokyo, Tokyo 113-8510, Japan

**Keywords:** point of care ultrasound, diaphragm, respiratory muscle, ageing

## Abstract

**Background/Objectives**: The effects of ageing on the diaphragm are unclear. This study examined the association between ageing and diaphragm thickness, thickening fraction (TF), and diaphragm excursion (DE) as assessed by ultrasonography after adjusting for other factors. The relationship between these parameters and maximal inspiratory pressure (MIP) was also investigated. **Methods**: From 2022 to 2024, ambulatory and communicative adult volunteers and outpatients were recruited from four Japanese medical institutions. Each participant’s background factors (including height, weight, and underlying diseases) and pulmonary function test results were assessed. Diaphragm thickness, TF, and DE were evaluated using ultrasonography. **Results**: The study involved 230 individuals with a mean age of 55.5 years (older adults (65 years and over), *n* = 117; non-older adults, *n* = 113). In older adults, the diaphragm was thicker (2.1 vs. 1.7 mm, *p* < 0.001), and TF was lower (88.7% vs. 116.0%, *p* < 0.001), with no significant difference in DE. Multivariate linear regression analysis adjusted for sex, height, body mass index, and underlying diseases showed positive associations between age and diaphragm thickness (*p* = 0.001), but not with TF or DE. MIP was positively associated with DE (*p* < 0.001) but not with thickness or TF. Age was negatively associated with MIP, regardless of diaphragm thickness, TF, and DE (all *p* < 0.001). **Conclusions**: As the diaphragm thickens with age, neither thickness nor TF is associated with MIP; only DE is related to MIP. Additionally, ageing is negatively associated with MIP, independent of diaphragm thickness, TF, and DE. Diaphragm function should be assessed using DE rather than thickness or TF.

## 1. Introduction

The diaphragm is the main respiratory muscle for inspiration. Reduced diaphragmatic function can decrease exercise tolerance and cause exertional dyspnoea. It can also weaken cough strength and increase gastroesophageal reflux, raising the aspiration risk [1,2,3]. The diaphragm also acts as a trunk muscle. Its contraction raises intra-abdominal pressure, aiding postural stability. This is crucial for older adults, who often experience reduced balance and limb strength. For them, the diaphragm helps to maintain posture and prevent falls [4,5]. Diaphragmatic dysfunction can be due to trauma-induced phrenic nerve damage, diaphragmatic muscle insufficiency or dysfunction in post-surgical patients, neurological disorders such as amyotrophic lateral sclerosis, critical illness polymyopathy, muscular diseases such as muscular dystrophy, and neuromuscular junction disorders such as myasthenia gravis and botulism [1,3]. Whether diaphragmatic function declines with age remains uncertain.

Diaphragm ultrasonography is a convenient method for assessing diaphragmatic function [1]. Key measurements include diaphragm thickness, the rate of thickness change during respiration (thickening fraction, TF), and diaphragmatic dome movement distance during respiration (diaphragm excursion, DE) [6]. Some reports show that diaphragm thickness is unaffected by ageing, while others suggest it may thin due to sarcopenia, as it is a skeletal muscle. Few studies have focused on the link between diaphragm thickness and ageing, and no consensus exists [4,7,8]. Moreover, few studies discuss the relationship between ageing and dynamic indices like TF and DE. Besides ageing, sex and body mass index (BMI) have been linked to diaphragm thickness and DE [6,8,9].

In older adults, sarcopenia is linked to reduced exercise capacity and increased fall and aspiration risks. Respiratory sarcopenia involves reduced respiratory muscle strength. Maximal inspiratory pressure (MIP) is key for evaluating it [10]. As the diaphragm is the main inspiratory muscle, reduced function may lower the MIP. Diaphragm ultrasonography shows a relationship between DE and MIP [9], but studies mainly involve younger people. This relationship in older adults remains unclear. This study controlled for factors like sex and BMI that affect ultrasonography results, enabling a precise examination of ageing’s link with diaphragm thickness, TF, and DE, and explored associations between diaphragm measures, MIP, and age.

## 2. Materials and Methods

### 2.1. Study Population and Setting

From March 2022 to January 2023, adult volunteers aged ≥18 years were recruited at Tokyo Medical and Dental University Hospital, Shinai Clinic, and Iizuka Hospital [6]. Additionally, starting in May 2023, results were collected over 1 year from ambulatory adult outpatients able to communicate, recruited at Tokyo Medical and Dental University Hospital, the Center for Health and Longevity, and Saiseikai Hitachi-Omiya Hospital. Inclusion criteria were consenting adults who could walk independently and follow instructions. Those with symptoms of acute infection (fever, respiratory distress), receiving home oxygen therapy, unable to communicate, or unable to complete all tests were excluded. Individuals aged ≥65 were defined as older adults. Participants provided sex, height, weight, and medical history information and then underwent pulmonary function tests and diaphragm ultrasonography. MIP was measured twice, with the higher value recorded. All procedures were performed by trained physicians or sonographers. Multiple hospitals and clinics were used to enhance diversity and generalisability. Standardised diaphragm ultrasonography procedures were used.

### 2.2. Ultrasound Measurements of Diaphragm

To measure the thickness of the right hemidiaphragm, a 7.0 MHz linear transducer was placed in a longitudinal and perpendicular orientation at the zone of apposition of the diaphragm, specifically near the eighth to ninth intercostal spaces along the anterior to mid-axillary line, with the participant in a seated position. The transducer was adjusted to bypass the ribs and ensure that a portion of the lung was visible on the screen’s edge during inspiration. At this location, the diaphragm thickness was recorded both at the peak of deep inspiration and at the end of expiration at functional residual capacity (FRC). These thickness measurements were performed using B-mode, marking from the midpoint of the echogenic line on the thoracic surface of the diaphragm to the midpoint of the line on the peritoneal surface. The TF was then determined using the following formula: TF = [(thickness at inspiration − thickness at expiration)/thickness at expiration] × 100. Both TF and DE were assessed during deep breathing. The DE for the right hemidiaphragm was evaluated around the eighth to ninth intercostal spaces from the anterior to mid-axillary line, an area in which the diaphragmatic dome is observable with the participant in a seated position. A 2.5 MHz phased-array transducer was aligned longitudinally and perpendicularly to the thoracic wall, with adjustments made to bypass the ribs. The M-mode scanning line was aligned to be as perpendicular to the diaphragmatic dome as feasible, capturing the variation in the dome’s positional change between inspiration and expiration.

### 2.3. Statistical Analysis

Comparisons of patient characteristics, diaphragm thickness, TF, and DE between older adults and non-older adults were conducted using the Wilcoxon rank-sum test for continuous variables and the chi-square test for categorical variables. Factors associated with each diaphragm ultrasonography outcome and MIP were examined using simple and multiple regression analyses. The model included age, sex, height, BMI, smoking history, and medical history as covariates. Participants who underwent ultrasound but did not provide test values were excluded from the statistical analysis. The coefficient, 95% confidence interval (CI), and *p*-value for each covariate were estimated. Statistical significance was defined as a *p*-value of <0.05. In previous studies, there has been no research comparing diaphragm function by age. As this is an exploratory study, the sample size was not calculated. All data were analysed with STATA software (version 17.0; StataCorp LLC., College Station, TX, USA).

## 3. Results

### 3.1. Participants

In total, 234 individuals were recruited, and four participants who could not undergo all tests were excluded. Data from 230 completed measurements were ultimately analysed. The participants’ characteristics are presented in Table 1. Among the 230 participants with complete assessments, 117 (50.8%) were older adults (aged ≥65 years). The mean Barthel index of the outpatient participants was 98.3 ± 4.2. Twelve (5.3%) participants had a history of stroke, all of whom had recovered with no residual paralysis or other sequelae.

### 3.2. Diaphragm Ultrasonography

The thickness of the right hemidiaphragm at FRC was measurable in all 230 participants, with an average thickness of 1.9 mm. Older adults had a significantly thicker diaphragm than non-older adults (2.1 vs. 1.7 mm, *p* < 0.001). The TF and DE during deep breathing were measurable in 228 and 212 participants, respectively. The main reason for the inability to measure these parameters was the diaphragm being obscured by the lungs during deep inspiration. The average TF was 102.3%, with non-older adults showing a significantly higher value (*p* < 0.001). The average DE was 4.4 cm, and there was no statistically significant difference between older and non-older adults (*p* = 0.772). The measurement results are presented in Table 2.

### 3.3. Relationship of Age with Diaphragm Thickness, TF, and DE: Simple and Multiple Regression Analyses

The results of the simple linear regression analysis of diaphragmatic thickness, TF, DE, and age are presented in Table 3. Age was significantly associated with diaphragmatic thickness and TF (both *p* < 0.001), with older age correlating with slightly increased diaphragmatic thickness and decreased TF. No significant association was found with DE (*p* = 0.665) (Supplementary Figure S1). Next, the results of the multiple linear regression analysis (adjusted for sex, height, BMI, and underlying diseases) for diaphragmatic thickness, TF, DE, and age are shown in Table 3. Age was positively associated with diaphragmatic thickness (*p* = 0.001), but not with TF or DE (*p* = 0.593 and *p* = 0.726, respectively). Female sex was negatively associated with DE (*p* = 0.001), and BMI was positively associated with diaphragmatic thickness and DE (both *p* < 0.001).

### 3.4. Relationship of MIP with Diaphragm Thickness, TF, and DE: Simple and Multiple Regression Analyses

A simple regression analysis was conducted to examine the association of MIP with diaphragm thickness, TF, and DE (Table 4). MIP and diaphragm thickness were measurable in 195 participants, TF in 193, and DE in 178. No statistically significant association was found between MIP and diaphragm thickness (*p* = 0.305). However, TF and DE showed statistically significant positive associations with MIP (*p* = 0.002 and *p* < 0.001, respectively) (Supplementary Figure S2). Next, a multiple regression analysis adjusted for age, sex, height, BMI, and underlying diseases was performed (Table 5). DE was positively associated with MIP (*p* < 0.001), indicating that greater DE was correlated with higher MIP. No statistically significant association of MIP with either diaphragm thickness or TF was found (*p* = 0.593 and 0.559, respectively). Instead, age, sex, BMI, and hypertension were found to have more significant associations.

## 4. Discussion

This study analysed the relationship between ageing, diaphragm ultrasonography, and MIP in 230 adults. Adjusted data showed that diaphragm thickness increased with age, but TF and DE were unaffected. Only DE was linked to MIP; diaphragm thickness and TF had no significant link to MIP. Ageing was independently associated with lower MIP, regardless of ultrasonography results.

### 4.1. Diaphragm Thickness and Ageing

In this study, the diaphragm was thicker in older than in non-older adults. The measurement of diaphragm thickness using ultrasonography is highly reproducible and shows a strong correlation with the thickness of the diaphragm measured directly through anatomical methods [10,11,12]. Generally, the skeletal muscle mass of the limbs increases until the age of approximately 30–40 years, after which it decreases, along with muscle function [13,14,15]. Both the abdominal muscles (such as the internal and external obliques) and the erector spinae muscles are also thinner in older adults [5,16]. Therefore, it is commonly assumed that the diaphragm, being a skeletal muscle, also becomes thinner and declines in function with ageing. However, previous studies have shown that diaphragm thickness is associated with factors such as sex and BMI [6,10,15] but not with ageing [15,17,18]. There are also reports indicating that the diaphragm is thicker in older than non-older adults [5,19], but a limitation of these studies is the small sample size. The present study had the largest sample size among such studies to date, and it showed that the diaphragm was thicker in older than in non-older adults, with diaphragmatic thickness gradually increasing with age. As mentioned above, diaphragm thickness is associated with factors such as sex and BMI [6,10,15]; even after adjusting for these factors, however, ageing was still identified as a factor contributing to increased diaphragm thickness.

The diaphragm is the primary respiratory muscle and plays a central role in respiratory homeostasis throughout life. It also has important functions in movement, muscle coordination, and posture maintenance [5,20]. In older adults, other trunk muscles may become thinner, potentially leading to compensatory hypertrophy of the diaphragm [5]. In addition, mouse models of early ageing exhibit diaphragm thickening due to pseudohypertrophy from muscle remodelling [21], a condition also observed in the early stages of human diseases, such as Duchenne muscular dystrophy [4,22]. These findings suggest that diaphragmatic thickening due to pseudohypertrophy may occur with ageing. Another possible cause of increased diaphragm thickness in older adults is infiltration of the muscle by fat. In the skeletal muscles of the limbs, fat infiltration increases with age, leading to an increase in intermuscular adipose tissue [23]. Therefore, the muscle strength per unit of muscle mass decreases with age, and maintaining muscle mass does not necessarily prevent a decline in muscle strength [23,24]. Additionally, the echogenicity of the diaphragm increases with age [15]. The reason for this increase in echogenicity is not yet clear. Generally, tissues with a higher fat content exhibit higher echogenicity, suggesting that the diaphragm in older adults may undergo increased fat infiltration. However, further research is needed to clarify this.

In summary, in older adults, compensatory hypertrophy due to the reduction in other trunk muscles, pseudohypertrophy secondary to ageing, and muscle fat infiltration may contribute to the increase in diaphragm thickness with age. This means that the thicker appearance of the diaphragm may represent a false increase in muscle mass without a corresponding functional improvement. The absence of an association between diaphragm thickness and MIP and the presence of a negative association between MIP and ageing support this notion.

### 4.2. TF, DE, and Ageing

In this study, univariate analysis showed a negative association between TF and ageing. After adjusting for age, sex, height, BMI, and underlying diseases, this association disappeared. Few studies have explored TF and ageing, but two found no link [6,15]. These studies had limitations like small sample size [15] and a low average age (~30 years), making it hard to assess ageing effects [6]. The present study had over double the sample size and included older adults (median age 65; range 28–78) yet found no link between TF and ageing. TF appears to be a universal indicator less affected by age, sex, or BMI. TF is used to diagnose diaphragmatic paralysis (TF <20%) [1], but whether this applies to older adults was unclear. Current results suggest this cutoff may be used across age groups.

DE showed no association with ageing in either the univariate or multivariate analysis. A study of the relationship between DE and ageing in 757 healthy individuals showed that DE (i.e., during deep breathing) increased with age beyond the age of 30 as follows: DE was 5.51 cm in those aged <30 years, 5.33 cm in those aged 31–50 years, 5.67 cm in those aged 51–65 years, and 6.10 cm in those aged >65 years [25]. However, this study did not adjust for factors that affect DE, such as BMI, making it difficult to determine the impact of ageing. Additionally, the Pearson correlation coefficient between age and DE was low at 0.164 [25]. The current evidence indicates that DE does not decrease with ageing and may either be not associated with ageing or even increase with ageing.

MIP and TF showed a positive association in the univariate analysis. However, after adjusting for age, sex, height, BMI, and underlying diseases, MIP was not associated with TF (*p* = 0.940). DE showed a positive association with MIP in both the univariate and multivariate analyses. Age was negatively associated with MIP independently of TF and DE. In a previous study examining the relationship between MIP, TF, DE, and ageing in 109 participants, MIP was not associated with TF; however, there was a positive association between DE and MIP. Age was also found to be associated with MIP independently of TF and DE [9]. Notably, the average age of the participants in this study was 32 years, so the findings provided only limited insight into the effects of ageing. In the present study, which included a sample size approximately twice that of the previous study and included older adults, the results were similar.

This study has several limitations. The participant group consisted of adult volunteers and patients recruited from outpatient clinics of medical institutions. There were more non-older adults among the volunteers and more older adults among the patients. Additionally, there were significantly more individuals with underlying diseases in the older adult group, suggesting that underlying diseases may have been a confounding factor. However, the mean Barthel index of the recruited patients was very high, indicating that the older adult group was largely independent despite the presence of underlying diseases. Given this, the study population can be considered to somewhat reflect the general population living in the community. Furthermore, a multivariate analysis was performed to account for the effects of confounders by including underlying diseases as an adjustment factor. Furthermore, this study focuses on Asian adults, and its applicability to minors or other ethnic groups remains unclear. Further research will be necessary to generalise these findings more broadly.

## 5. Conclusions

In summary, ageing is an independent factor positively associated with increased diaphragm thickness. However, ageing is not related to TF or DE. Even with increased diaphragm thickness, there is no association with MIP; in diaphragm ultrasonography, only DE is associated with MIP. Ageing is negatively associated with MIP, independent of diaphragm thickness, TF, and DE. TF is less influenced by various factors and could serve as a universal indicator applicable to both younger and older individuals, making it potentially useful as a criterion for diagnosing diaphragmatic paralysis. However, from the perspective of MIP, DE should be used to evaluate diaphragm function with ultrasonography.

## Figures and Tables

**Table 1:** Participant characteristics.

Characteristics	Total (*n* = 230)	Older Adults (*n* = 117)	Non-Older Adults (*n* = 113)	*p*-Values **
Age (years)	55.5 ± 24.9	78.1 ± 6.9	32.2 ± 11.8	<0.001 *
Male sex	110 ± 47.8	52 ± 44.4	58 ± 51.3	0.296
Height (cm)	160.4 ± 10.2	155.6 ± 8.5	165.4 ± 9.5	<0.001 *
Body mass index (kg/m^2^)	22.8 ± 3.1	23.4 ± 3.3	22.1 ± 2.8	<0.001 *
%VC	95 ± 14.5	93.7 ± 17.6	96.2 ± 10.4	0.337
FEV_1_/FVC	81.7 ± 7.7	78.1 ± 7.9	85.5 ± 5.6	<0.001 *
Smoking history:				
Never smoker	167 (72.6)	72 (61.5)	95 (84.1)	<0.001 *
Current smoker	11 (4.8)	7 (6.0)	4 (3.5)	
Former smoker	52 (22.6)	38 (32.5)	14 (12.4)	
Medical history:				
Hypertension	70 (30.6)	68 (58.1)	2 (1.8)	<0.001 *
Dyslipidaemia	67 (29.3)	67 (57.3)	0 (0.0)	<0.001 *
Diabetes mellitus	67 (29.3)	67 (57.3)	0 (0.0)	<0.001 *
COPD	5 (2.2)	4 (3.4)	1 (0.9)	0.188
Stroke	12 (5.3)	12 (10.3)	0 (0.0)	<0.001 *
Charlson comorbidity index	0.8 (1.3)	1.6 (1.5)	0 (0.0)	<0.001 *

Data are presented as mean ± standard deviation or *n* (%). Older adults: ≥65 years of age. Non-older adults: <65 years of age. * *p* < 0.05. ** Comparison between older adults and non-older adults. The *p*-values were estimated by the Wilcoxon rank-sum test for continuous variables or the chi-square test for categorical variables. COPD, chronic obstructive pulmonary disease; FEV1, forced expiratory volume in 1 s; FVC, forced vital capacity; %VC, percentage of vital capacity.

**Table 2:** Diaphragm thickness, thickening fraction, and excursion of right diaphragm.

Parameters	Total	Older Adults	Non-Older Adults	*p*-Values **
Diaphragm thickness at FRC (mm, *n* = 230)	1.9 ± 0.5	2.1 ± 0.6	1.7 ± 0.4	<0.001 *
Thickening fraction (%, *n* = 228)	102.3 ± 48.6	88.7 ± 44.2	116.0 ± 49.2	<0.001 *
Diaphragm excursion (cm, *n* = 212)	4.4 ± 1.4	4.4 ± 1.4	4.4 ± 1.4	0.772

Data are presented as mean ± standard deviation. Older adults: ≥65 years of age. Non-older adults: <65 years of age. * *p* < 0.05. ** Comparison between older and non-older adults. The *p*-values were estimated using the Wilcoxon rank-sum test. FRC, functional residual capacity.

**Table 3:** Results of simple and multiple regression analyses for right hemidiaphragm thickness (mm), thickening fraction (%), and diaphragm excursion.

Independent Variables	Outcome Variables					
	Diaphragm Thickness at FRC (*n* = 230)		Thickening Fraction (*n* = 228)		Diaphragm Excursion (*n* = 212)	
Parameters	Coefficients (95% CI)	*p*-Values	Coefficients (95% CI)	*p*-Values	Coefficients (95% CI)	*p*-Values
**Simple regression**						
Age	0.01 (<0.01, 0.01)	<0.001 *	−0.49 (−0.73, −0.24)	<0.001 *	0.02 (−0.06, 0.09)	0.665
**Multiple regression (age, adjusted for independent variables)**						
Age	0.01 (<0.01, 0.01)	0.001 *	−0.11 (−0.54, 0.31)	0.593	0.02 (−0.09, 0.13)	0.726
Female sex						
(reference: male)	−0.01 (−0.21, 0.19)	0.897	1.44 (−18.29, 21.12)	0.886	−8.70 (−13.96, −3.45)	0.001 *
Height	0.01 (−0.01, 0.02)	0.317	0.62 (−0.44, 1.68)	0.247	0.26 (−0.02, 0.55)	0.072
Body mass index	0.04 (0.02, 0.06)	<0.001 *	−0.19 (−2.31, 1.94)	0.863	1.41 (0.81, 2.01)	<0.001 *
Smoking history **	−0.01 (−0.18, 0.16)	0.871	0.33 (−16.28, 16.95)	0.969	−3.64 (−8.10, 0.80)	0.107
Hypertension	0.11 (−0.08, 0.30)	0.248	−19.86 (−38.37, −1.36)	0.035 *	0.50 (−4.34, 5.34)	0.839
Dyslipidaemia	−0.12 (−0.31, 0.07)	0.215	0.61 (−18.42, 19.65)	0.950	2.19 (−2.67, 7.05)	0.375
Diabetes mellitus	−0.02 (−0.21, 0.17)	0.813	−0.02 (−20.76, 16.73)	0.832	1.75 (−3.02, 6.53)	0.470
COPD	0.44 (−0.02, 0.90)	0.059	−11.54 (−56.57, 33.49)	0.614	0.77 (−11.96, 13.50)	0.905
Stroke	0.11 (−0.19, 0.41)	0.477	−1.04 (−30.62, 28.54)	0.945	−4.58 (−12.41, 3.26)	0.251

* *p* < 0.05. ** Current or former smoking history. CI, confidence interval; FRC, functional residual capacity; COPD, chronic obstructive pulmonary disease.

**Table 4:** Results of simple regression analysis for MIP and each diaphragm measurement.

Independent Variables	Outcome Variable: MIP	
Parameters	Coefficients (95% CI)	*p*-Values
Diaphragm thickness (FRC, *n* = 195)	−4.05 (−11.82, 3.71)	0.305
Thickening fraction (*n* = 193)	0.12 (0.05, 0.20)	0.002 *
Diaphragm excursion (*n* = 178)	0.51 (0.23, 0.79)	<0.001 *

CI, confidence interval; FRC, functional residual capacity; MIP, maximal inspiratory pressure. * *p* < 0.05.

**Table 5:** Results of multiple regression analysis for MIP and result of each diaphragm measurement adjusted for independent variables.

Independent Variables	Outcome Variable: MIP					
	Model 1 (*n* = 195)		Model 2 (*n* = 193)		Model 3 (*n* = 178)	
Parameters	Coefficients (95% CI)	*p*-Values	Coefficients (95% CI)	*p*-Values	Coefficients (95% CI)	*p*-Values
Diaphragm thickness (FRC)	1.74 (−4.67, 8.15)	0.593				
Thickening fraction			0.02 (−0.04, 0.08)	0.559		
Diaphragm excursion					0.43 (0.19, 0.67)	<0.001 *
Age	−0.56 (−0.75, −0.37)	<0.001 *	−0.55 (−0.74, −0.36)	<0.001 *	−0.58 (−0.77, −0.39)	<0.001 *
Female sex (reference: male)	−12.63 (−21.29, −3.97)	0.004 *	−12.79 (−21.47, −4.11)	0.004 *	−5.85 (−15.10, 3.40)	0.214
Height	0.24 (−0.23, 0.71)	0.311	0.24 (−0.23, 0.72)	0.319	0.28 (−0.22, 0.77)	0.267
Body mass index	2.26 (1.25, 3.26)	<0.001 *	2.34 (1.40, 3.28)	<0.001 *	1.66 (0.56, 2.76)	0.003 *
Smoking history **	−4.90 (−12.10, 2.30)	0.181	−4.63 (−11.88, 2.62)	0.209	−0.94 (−8.43, 6.55)	0.805
Hypertension	−9.71 (−18.24, −1.17)	0.026 *	−9.59 (−18.33, −0.85)	0.032 *	−10.48 (−19.20, −1.77)	0.019 *
Dyslipidaemia	0.93 (−8.53, 10.38)	0.847	0.32 (−9.35, 9.99)	0.948	1.28 (−8.06, 10.61)	0.787
Diabetes mellitus	−0.43 (−10.23, 9.36)	0.931	0.09 (−10.00, 10.18)	0.986	−0.40 (−10.04, 9.24)	0.935
COPD	−18.84 (−39.41, 1.73)	0.072	−18.31 (−38.82, −2.19)	0.08	−14.51 (−37.19, 8.15)	0.208
Stroke	0.26 (−16.42, 19.64)	0.976	0.94 (−15.79, 17.68)	0.912	3.94 (−12.50, 20.39)	0.637

Model 1: Regression analysis model of MIP and thickness adjusted for age, sex, height, body mass index, and underlying diseases. Model 2: Regression analysis model of MIP and thickening fraction adjusted for age, sex, height, body mass index, and underlying diseases. Model 3: Regression analysis model of MIP and diaphragm excursion adjusted for age, sex, height, body mass index, and underlying diseases. * *p* < 0.05. ** Current or former smoking history. CI, confidence interval; COPD, chronic obstructive pulmonary disease; FRC, functional residual capacity; MIP, maximal inspiratory pressure.

## Data Availability

The data presented in this study are available on request from the corresponding author due to the sensitivity of the data.

## Supplementary Materials

The following supporting information can be downloaded at: https://www.mdpi.com/article/10.3390/diagnostics15020163/s1, Figure S1: Scatter plot and regression line of diaphragm thickness, thickening fraction, diaphragm excursion, and age; Figure S2: Scatter plot and regression line of diaphragm thickness, thickening fraction, diaphragm excursion, and maximal inspiratory pressure.

## Abbreviations

The following abbreviations are used in this manuscript:

BMI	Body mass index
DE	Diaphragm excursion
FRC	Functional residual capacity
MIP	Maximal inspiratory pressure
TF	Thickening fraction
