# Supplementary material for: The Impact of Ageing on Diaphragm Function and Maximal Inspiratory Pressure: A Cross-Sectional Ultrasound Study
Source: Diagnostics (Basel). 2025 Jan 13;15(2):163. doi: 10.3390/diagnostics15020163 (PMC11763467; doi:10.3390/diagnostics15020163)
Supplement: Supplementary file 1 [file diagnostics-15-00163-s001.zip › diagnostics-3419541-supplementary.pdf]

Supplementary figures

The impact of ageing on diaphragm function and maximal inspiratory pressure: A cross-sectional ultrasound study

Toru Yamada, Taro Minami, Takahiro Shinohara, Shuji Ouchi, Suguru Mabuchi, Shumpei Yoshino, Ken Emoto, Kazuharu Nakagawa, Kanako Yoshimi, Mitsuko Saito, Ayane Horike, Kenji Toyoshima, Yoshiaki Tamura, Atsushi Araki, Ryoichi Hanazawa, Akihiro Hirakawa, Takeshi Ishida, Takuma Kimura, Haruka Tohara, and Masayoshi Hashimoto

Supplementary Figure S1 Scatter plot and regression line of diaphragm thickness, thickening fraction, diaphragm excursion, and age.

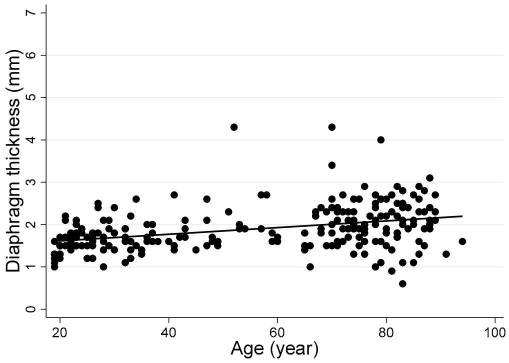

(a)

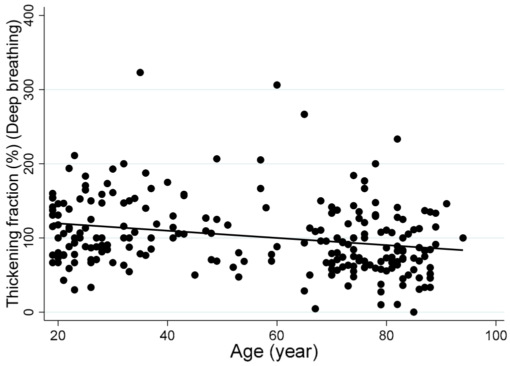

(b)

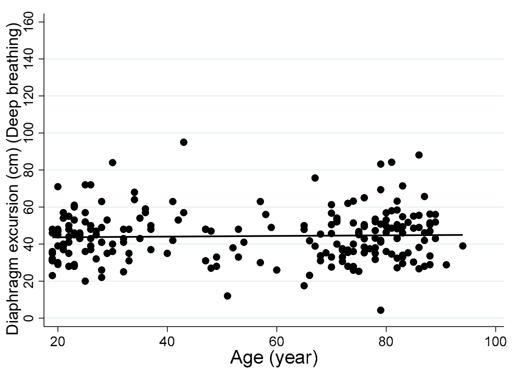

(c)

(**a**) Scatter plot and regression line: diaphragm thickness and age. Regression line: Y=0.01X+1.45, p<0.001. (**b**) Scatter plot and regression line: Thickening fraction and age. Regression line: Y=−0.49X+129.21, p<0.001. (**c**) Scatter plot and regression line: Diaphragm excursion and age. Regression line: Y=0.02X+43.39, p=0.665. *The black line in each figure represents the regression line. The coefficient and p-value were estimated by simple regression analysis.

Supplementary Figure S2 Scatter plot and regression line of diaphragm thickness, thickening fraction, diaphragm excursion, and maximal inspiratory pressure.

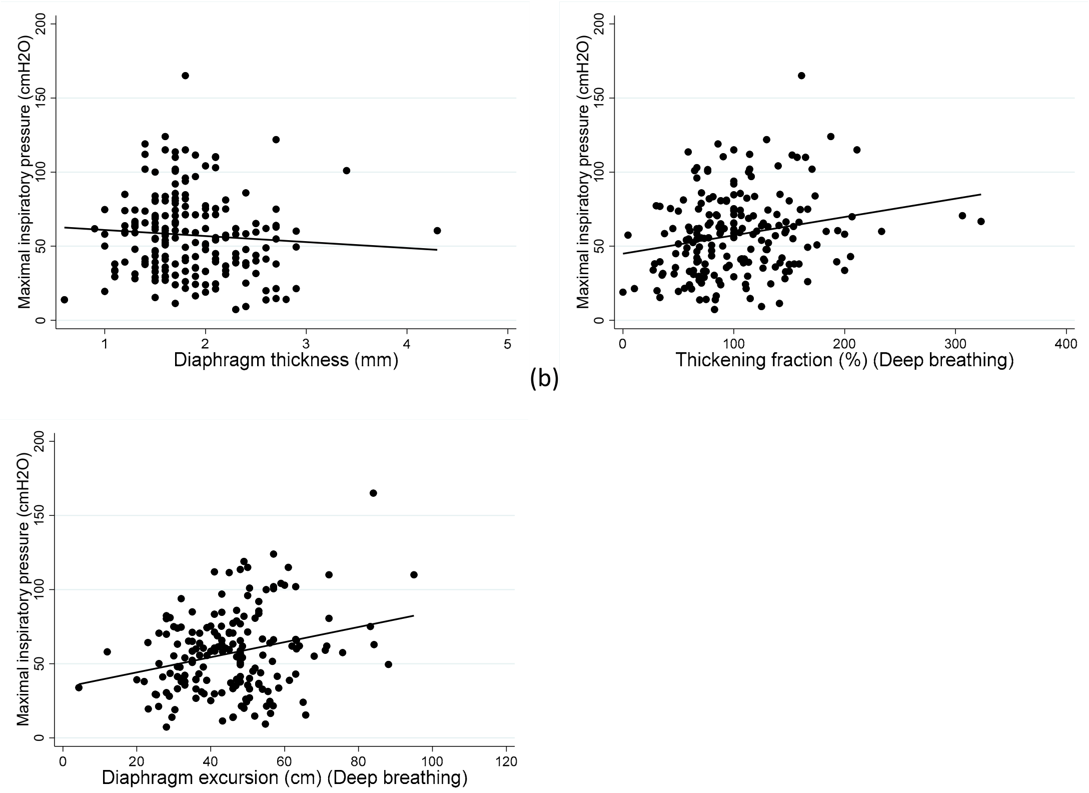

(a) (b) (c)

(**a**) Scatter plot and regression line: Diaphragm thickness and maximal inspiratory pressure. Regression line: Y=−4.05X+64.98, p=0.305. (**b**) Scatter plot and regression line: Thickening fraction and maximal inspiratory pressure. Regression line: Y=0.12X+45.01, p=0.002. (**c**) Scatter plot and regression line: Diaphragm excursion and maximal inspiratory pressure. Regression line: Y=0.51X+33.95, p<0.001. *The black line in each figure represents the regression line. The coefficient and p-value were estimated by simple regression analysis.
